# Effects of Storage Moisture Content on Corn Stover Biomass Stability, Composition, and Conversion Efficacy

**DOI:** 10.3389/fbioe.2020.00716

**Published:** 2020-07-14

**Authors:** William A. Smith, Lynn M. Wendt, Ian J. Bonner, J. Austin Murphy

**Affiliations:** Idaho National Laboratory, Idaho Falls, ID, United States

**Keywords:** biomass, dry matter loss, aerobic storage, feedstock logistics, composition, sugar release

## Abstract

Variable moisture content of biomass during storage is known to compromise feedstock stability, yet a great deal of uncertainty remains on how to manage or mitigate the issue. While moisture contents above 20% risk unacceptable losses in aerobic feed and forage storage, no quantitative relationship exists between corn stover moisture content and rates or extents of degradation for bioenergy use. This work quantifies the relationship between initial moisture content of aerobically stored corn (*Zea mays* L.) stover biomass and dry matter loss through time. Corn stover with 20% to 52% moisture was stored under aerobic conditions in laboratory reactors while dry matter loss was measured in real time, reaching extents of 8% to 28% by the end of storage. Rates and extents of degradation were proportional to moisture content but were not linearly related. A moisture content “threshold” exists between 36% and 52% above which rates and extents of degradation increase rapidly. Compositional changes included glucan and lignin enrichment resulting from hemicellulose component (xylan and acetyl) biodegradation. Moisture desorption characteristics of the post-storage materials suggest chemical and/or physical changes that increase biomass hydrophilicity. Monomerization of carbohydrates though dilute acid pretreatment and enzymatic hydrolysis resulted in only minor changes, suggesting that degradation does not negatively influence conversion potential of the remaining biomass. Total dry matter preservation remains one of the most significant challenges for a biorefinery.

## Introduction

Corn (*Zea mays* L.) stover has been targeted as an early adoption feedstock for pioneer biofuel production in the United States because of its current availability and low cost (Hess et al., 2009). However, use of agricultural residues challenges the ability to control biomass quality as harvest timing and operations are dictated by the primary crop. Because stover biomass is not the highest priority during grain harvest, variations in biomass conditions, specifically moisture content, can be large within a given year and between years (Kenney et al., 2013). Corn stover moisture contents at harvest are predicted to exceed 40% (wet basis) nationwide over approximately one third of the United States in an average year (Oyedeji et al., 2017). Moisture contents >20% threaten the stability of aerobically stored feedstock (Darr and Shah, 2014). For a year-round conversion facility dependent on a seasonally available feedstock, uncertainty of losses throughout long term storage can have serious consequences relative to operational efficiency and costs at the biorefinery (Rentizelas et al., 2009; Darr and Shah, 2014).

Numerous research efforts have demonstrated the volatility of dry matter loss of biomass stored in various configurations and across a range of moisture contents (McGechan, 1990; Shinners et al., 2007, 2010, 2011; Shah et al., 2011; Smith et al., 2013). However, because of the inherent challenges related to environmental control, sampling, and replication of field-scale storage studies, no conclusive relationships have been developed to describe the rates and extents of dry matter loss in a way that enables proactive decision making throughout a year-long storage period. Without a functional understanding of biomass losses during storage it is difficult to accurately assess the quantity of feedstock that must be procured and how that supply should be handled throughout the year. To combat this, analyses of feedstock logistics systems typically assume users will over-purchase to ensure that an adequate supply of biomass is on hand to cover a generalized – or assumed “average” – dry matter loss (Rentizelas et al., 2009). While this method is effective for “average” years, it does not account for instances beyond the norm. Considering the severe droughts and flooding events that have impacted corn production in the United States Midwest over the past several years, an understanding of “abnormal” conditions on feedstock logistics is prudent and necessary.

The costs for uncertain losses in storage impact a range of stakeholders within a bioenergy production system. In cases where on-farm storage is employed and payment for the biomass is conducted at the time of delivery to a biorefinery, the farmer incurs the direct financial consequence of storage losses. In this case, the farmer invested in the harvest, collection, and storage of an initial mass of material, but was only able to deliver and be paid for the original mass less dry matter losses, effectively inflating their production costs and reducing their profit. Two primary concerns arise in this scenario: (1) how does this reduced return impact grower satisfaction and continued participation in biomass production, and (2) is the end-user prepared to source additional material (presumably from greater distances and at greater costs) to offset the losses suffered in storage? On the other hand, if centralized or satellite storage is used in a way where the refinery owns the biomass immediately after harvest, losses during storage effectively increase the end-users realized feedstock price. For example, if feedstock was purchased at a farm-gate price of 40 $ tonne^–1^ and the material suffered 10% loss in storage before being used, the as-recovered material is being consumed at price of 44.4 $ tonne^–1^. This represents a 10% loss in revenue for the producer/farmer. For end-users processing hundreds of thousands of tonnes per year, such cost increases and uncertainties in available inventory can be large, the number of contracts necessary to provide enough material can increase, the supply radius required to source the materials will grow, and final fuel selling prices may be negatively impacted (Cafferty et al., 2013). Regardless of who owns the biomass during the period of degradation, the consequences of material loss and quality changes in storage will negatively impact both the producer—less/lower quality material delivered—and the biorefinery—lower quality material and a need to purchase additional material to replace the lost/degraded biomass. Because of this, we must understand how biomass supply systems can operate in the face of uncertain conditions, how these systems can adapt to natural variation, and ultimately how this variability impacts the costs of procuring biomass and producing renewable fuels.

Since high moisture corn stover (>30% at grain harvest) will occur in areas that typically produce “dry” stable biomass (Oyedeji et al., 2017), methods of mitigating the risks associated with storage losses are needed. As discussed by Darr and Shah (2014), a number of different storage methods may be employed to improve the stability of biomass in storage, with anaerobic methods being the common and trusted option for stabilizing feedstocks of exceedingly high moistures, and protection from precipitation being the go-to for ensuring stability of dry materials. However, even when following conventional best management practices, risk and uncertainty in storage remain, as demonstrated by Smith et al. (2013), who showed plastic wrapped high moisture bales of energy sorghum to reach over 40% dry matter loss by 9 months in storage and tarped low moisture sorghum to suffer 25% dry matter loss over the same time. Rentizelas et al. (2009) proposed an alternative management solution utilizing multiple feedstocks harvested at different points throughout the year to minimize the total time spent in storage. While the researchers concluded that a multi-feedstock approach was effective, differences in the cost and benefit of various storage methods failed to outcompete low cost storage solutions for low value biomass. As a result, pioneer biorefineries have had to struggle with feedstock storage related challenges such as variations in moisture, material composition, and yield (Lamers et al., 2015).

Dry matter losses in stored biomass occur when microorganisms use available carbohydrates for growth and energy. Losses not only result in a reduction of biomass quantity, they also result in biomass that is compositionally altered because the microorganisms consume both soluble and structural sugars, leaving behind the more recalcitrant structural sugars as well as enriching the biomass in lignin and ash (Shinners et al., 2011; Smith et al., 2013). Previous research has shown a relative decrease in xylan and increase in glucan percentages in corn stover that experienced high levels of dry matter loss as a result of preferential hemicellulose degradation (Wendt et al., 2014, 2018). While the combined structural sugar content of the recovered dry matter remains high, the reactivity of the remaining structural sugars relative to the starting material is uncertain without additional conversion testing. Few comparative studies exist that show the impact of storage losses on conversion performance of herbaceous biomass (Agblevor et al., 1994, 1996) and none to date describe the impacts of dry matter loss on conversion performance in dilute-acid pretreatment and enzymatic hydrolysis.

The microbial activity that drives carbohydrate loss under aerobic conditions is primarily controlled by reducing moisture content in storage (Shinners et al., 2007). However, the water activity rather than the water content of the material directly affects the rates and extents of biodegradation (Beuchat, 1983). Water activity (*a*_*w*_) can be defined functionally as the relative humidity of the air around a material that is at its equilibrium moisture content at any given temperature. A water activity between 0.6 and 0.7 represents the lower bound at which most bacterial and fungal activity may occur (Beuchat, 1981), thus represents a biologically stable storage state. Water contents (wet basis; abbreviated w.b.) for corn stover biomass fractions at 0.6 to 0.7 *a*_*w*_ range from 12% to 14% (Igathinathane et al., 2005). Rates and extents of biodegradation increase as water activities go from 0.6 (osmophilic yeasts), through 0.8 (most molds), and beyond 0.9 (most bacteria) (Beuchat, 1981). However, the relationship between water activity and biodegradation is not necessarily linear nor easily estimated since biodegradation is also dependent on substrate availability, temperature, and the composition and functional abilities of the microbial communities initially present on the substrate. For complex substrates such as corn stover, each plant tissue type may have its own water content/water activity relationship (Igathinathane et al., 2007). The net result is dependent on the sum of the parts, thus will vary as the tissue composition changes as result of what tissues are there and how they change as a result of biodegradation.

The objectives of this work are to: (1) quantify the rates and extents of corn stover biodegradation occurring at a range of fixed moisture contents, (2) measure the change in biomass chemical components (structural and soluble sugars, lignin, and ash) resulting from dry matter loss, (3) measure the change in reactivity to pretreatment and enzymatic hydrolysis resulting from dry matter loss, and (4) begin to develop the parameters needed to predict biomass storage stability for aerobically stored corn stover. This work uses laboratory reactors to monitor corn stover biomass under aerobic conditions and at 20%, 25%, 30%, 36%, and 52% moisture contents (w.b) and measure dry matter loss in real-time. Rates and extents of biodegradation by native microflora are compared for each condition along with the corresponding compositional changes. Reactivity, measured by sugar yield after pretreatment and subsequent enzymatic hydrolysis, was evaluated to understand the impact on biorefinery conversion potential. Finally, adsorption isotherms of the stored materials were used to evaluate the water content and water activity relationships between native and degraded materials. The data generated by this work provides the foundation for understanding the relationship between biomass moisture content in storage, storage stability, and the resultant impact of biodegradation on the as-delivered biomass composition. These relationships are needed to predict biomass storage performance relative to harvest timing/moisture content, storage duration/delivery scheduling, and material blending to reduce day-to-day quality variations.

## Materials and Methods

### Biomass and Material Preparation

Corn stover was harvested using an AGCO LB34B single-pass baler in Stevens County, KS, at a moisture content of 55%. A portion of the biomass was immediately packed into 208 L drums, transported to Idaho National Laboratory (INL), Idaho Falls ID, and stored at −20°C. Prior to initiating the experiments, each drum of material (approximately 50 kg of wet biomass) was spread into a thin-layer to thaw and dry to a specified moisture content at ambient room temperature (17° to 23°C). A total of five drums of material was used to provide material at 20%, 25%, 30%, 36%, and 52% moisture (Table 1). Each lot of material was homogenized and split by hand before being loaded into duplicate reactors for storage.

**TABLE 1:** Corn stover moisture content, load mass (dry mass), and dry matter density for each of the ten storage reactors.

Sample ID	Moisture Content,% wb	Initial Load Mass, kg	Dry Bulk Density, kg/m^3^
20A	20.1	7.3	76.3
20B	20.1	8.3	87.5
25A	25.9	7.9	76.9
25B	25.9	9.3	90.3
30A	30.6	8.5	77.9
30B	30.6	9.8	89.8
36A	36.5	11.0	91.9
36B	36.5	12.1	100.8
52A	52.2	15.9	99.7
52B	52.2	16.9	105.9

### Laboratory Storage

The design and function of the laboratory scale storage reactors used in this work have been described by Bonner et al. (2015) and applied to corn stover storage research by Wendt et al. (2014). Each reactor consists of a 100 L inner chamber (76 L usable) for housing a biomass sample (approximately 8 to 10 kg dry matter) surrounded by a temperature-controlled water jacket. A feedback loop between a LabVIEW (Version 11.0.1, National Instruments, Austin, TX, United States) control interface and temperature sensors in the biomass column is used to control the jacket temperature to 0.5°C below that of the biomass in the center of the reactor, allowing natural biological self-heating to drive the storage temperature with minimal heat loss to the chamber walls. Atmospheric air is supplied via mass flow controller (Brooks Inst. Model 5850E, Hatfield, PA, United States) at 1 L min^–1^ into the bottom of the reactor where it is heated and humidified by bubbling through a 7.6 cm layer of water before flowing upward through the biomass column and out a single port at the top of the reactor. Gas exiting the reactor is then routed through a glycol chilled condensation coil to remove moisture prior to automated sampling and delivery to a gas chromatograph (Agilent MicroGC300, Santa Clara, CA, United States) for oxygen, nitrogen, and carbon dioxide analyses every 4 h.

Corn stover at each of the five moisture contents was tested in duplicate, requiring a total of ten reactor runs. Biomass was packed into the reactors by hand at a dry matter density of 90 ±10 kg m^3^ (Table 1), which is similar to other reported studies (Wendt et al., 2014, 2018; Bonner et al., 2015).

Once in operation, reactors were allowed to self-heat naturally while the temperature of the column and the composition of the gas exiting each reactor were recorded. Reactor trials were terminated once both duplicates had returned to and stabilized at ambient room temperatures (23° to 25°C), which corresponded to a bulk respiration rate of <0.4 g CO_2_ (kg DM remaining)^–1^ d^–1^ and resulted in several days difference between completion times for individual reactors, which ran from 55 to 85 days. The biomass from each reactor was unloaded individually, homogenized by mixing in a new 3-mil food-grade super-sack liner (BAG Corp, Richardson, TX, United States) for each reactor. Material was spread to a depth of 5 to 10 cm within the sack liner. Composite samples of 100 to 125 g (fresh) mass were randomly collected (*n* = 3) and used for analyses.

### Material Analysis

Moisture content of the biomass before and after storage was measured by drying a subsample at 105°C for 24 h. Dry matter loss of each reactor was determined by utilizing the CO_2_ concentration measured in the off-gas during storage to calculate the consumption of carbohydrate (CH_2_O) though aerobic respiration using a molar ratio of 1:1 (McGechan, 1989), such that:

(1) $$\mathrm{Dry}\mathrm{Matter}\mathrm{Loss}\left(\%\right)=\frac{\sum {\mathrm{CH}}_{2}\,O}{{\mathrm{DM}}_{i}}\cdot 100$$

Where the cumulative mass of CH_2_O at any time in storage is related to the initial dry mass, DM_i_ to calculate dry matter loss over the entire storage period.

Chemical compositional analysis of unstored and stored corn stover composite samples was performed in duplicate according to standard biomass procedures developed by the National Renewable Energy Laboratory (NREL) (Sluiter and Sluiter, 2011). Extractives from water and ethanol were determined using an ASE 350 (Dionex, Sunnyvale, CA, United States) (Sluiter et al., 2008b). The extracted biomass was subjected to a two-stage acid hydrolysis (Sluiter et al., 2008a). The liquor from the acid hydrolysis was analyzed using HPLC with a refractive index detector for monomeric sugars and UV-VIS (210 nm) sugar degradation products (Agilent, Santa Clara, CA, United States) and an Aminex HPX 87P and 87H columns (Bio-Rad, 300 × 7.8 mm, Hercules, CA, United States). The solids were used to determine lignin and ash (Sluiter et al., 2008a). Acid-soluble lignin fractions were analyzed using a Varian Cary 50 ultraviolet-visible spectrometer (Agilent, Santa Clara, CA, United States) (Sluiter et al., 2008b). The compositional analysis of unstored corn stover was performed on a sample from each moisture content and averaged (*n* = 5), while duplicate reactors were averaged for each moisture condition.

Organic acids were extracted from the unstored and stored corn stover composite samples in duplicate using a 1:10 ratio of wet biomass (50 g) to 18 MΩ-cm nanopure water. Samples equilibrated at 4°C for 72 h. An aliquot was filtered to 0.2 μm and acidified to a pH of 4 with sulfuric acid. Organic acids were analyzed using high performance liquid chromatography (HPLC) with a refractive index detector (Waters, Milford, MA, United States) and an Aminex HPX 87H ion exclusion column (Bio-Rad, 300 × 7.8 mm, Hercules, CA, United States).

### Pretreatment and Enzymatic Hydrolysis

Dilute acid pretreatment was performed using a Dionex ASE 350 Accelerated Solvent Extractor (Dionex Corporation, Sunnyvale, CA, United States) at 10% (w/w) solids loading by adding 30 mL of 1% sulfuric acid (w/w) in 66-mL Dionium cells, as described previously (Wolfrum et al., 2013). Briefly, reaction conditions included a 360 s ramp in temperature to 130°C followed by a 420 s incubation (severity factor = 1.73), which were determined to be optimal for corn stover by Wolfrum et al. (2013). A 150 mL rinse was then performed at 100°C to neutralize the biomass. The pretreatment liquor was analyzed for monomeric and polymeric sugars using HPLC with the HPX-87P column, as described above. Fermentation inhibitors including acetate, furfural, 5-hydroxymethylfurfural, and levulinic acid were measured using HPLC with the HPX-87H column, as described above. Yields were calculated based on glucan and xylan levels in the initial biomass sample compared to glucan and xylan released during pretreatment. For both pretreatment and enzymatic hydrolysis experiments, all feedstock composition, and hydrolyte liquors organic acid, monomeric sugar, and total sugar concentrations were determined using appropriate National Renewable Energy Laboratory (NREL) laboratory analytical procedures (LAPs) (Sluiter et al., 2008a, b; Sluiter and Sluiter, 2011), which include the yield calculations. Triplicate pretreatment experiments were conducted on duplicate samples for each storage treatment, and the results were combined (*n* = 6).

Enzymatic hydrolysis was performed in triplicate on non-pretreated and pretreated, washed biomass. Briefly 0.15 g (dry basis) of biomass was added to a 20 ml scintillation vial at 1.5% (w/w) solids loading and 50 mM sodium citrate buffer, pH 4.8, based on methods from Selig et al. (2008) and used in our laboratory by Hoover et al. (2018). Final reaction volume was 10 mL. Cellic^®^ CTec2 and HTec2 enzyme complexes (Novozymes; Franklinton, NC, United States) were added at loading rates of 20 mg protein and 2 mg protein per g dry mass biomass, respectively. Sodium citrate buffer was supplemented with 0.02% NaN_3_ in the biomass slurry to prevent microbial contamination. Enzyme and substrate blanks were prepared as controls. After an incubation period of 120 h at 50°C (New Brunswick Innova 4080, Enfield, CT, United States), aliquots of liquor were removed, filtered through a 0.2 μm filter, and analyzed for monomeric sugars using Megazyme assay kits (D-Glucose GOPOD Format Kit for glucose, D-Xylose Assay Kit for xylose; Bray, Ireland). Sugar yields were calculated by dividing the sugar released in dilute-acid pretreatment and enzymatic hydrolysis liquors by the initial sugar content in the biomass sample. Reactivity in pretreatment, enzymatic hydrolysis, and combined pretreatment and enzymatic hydrolysis was calculated by dividing the sum of released and xylan released based on the total glucan and xylan present in the hydrolysis product from the initial materials’ compositional analyses.

## Material Isotherms

Water activity isotherms were generated using a Decagon Devices Inc., AquaSorp Isotherm Generator (Pullman, WA, United States). The instrument uses a dynamic dew-point isotherm method, which unlike traditional salt-slurry isotherm methods, automatically records the sample’s mass and water activity over time as it is exposed to desiccant dried or water saturated air, causing the sample to undergo desorption or adsorption, respectively (Schmidt and Lee, 2012). The instrument operates at a fixed temperature (0.1°C) with an internal micro-balance (0.1 mg) and chilled mirror dew-point sensor (0.005 *a*_*w*_), eliminating the need to manually handle the sample and disrupt the testing conditions. Cycling isotherms consisting of an initial desorption, adsorption, and second desorption were recorded from 0.05 *a*_*w*_ to 0.85 *a*_*w*_ (or 5% to 85% equilibrium relative humidity or *e.m.c*). The instrument’s sample cup was loaded with 250 mg to 500 mg of material ground to pass a 2 mm screen (Thomas Model 4 Wiley mill, Thomas Scientific, Swedesboro, NJ, United States) and equilibrated to the test temperatures within the sealed sample chamber before desorption began.

Tests were performed at 25°C with triplicate samples of the starting material and duplicate samples of the stored materials (one composite sample from each reactor). The instrument’s airflow over the sample material was set to 60 mL/min. Completed samples were dried at 105°C for 24 h to determine dry mass for calculating moisture content.

Isotherms were fit to the temperature-independent GAB model [Guggenheim-Anderson-de Boer; van den Berg and Bruin (1981)] using Decagon SorpTrac software (v. 1.14; Decagon Devices Inc., Pullman, WA, United States). Model calculations are presented in equation 2, below. The GAB model is widely used in the food industry and its parameters relate to material specific properties, where *C* is a heat (energy) constant, *k* is a material specific drying parameter, and *M*_0_ is the monolayer moisture content (expressed in the dry basis).

(2) $$e.m.c=\frac{M_{0}\cdot k\cdot C\cdot a_{w}}{\left[\left(1-k\cdot a_{w}\right)\,\left(1-k\cdot a_{w}+C\cdot k\cdot a_{w}\right)\right]}$$

The monolayer moisture content is the moisture content at which all hydrophilic groups present in a material are associated with a water molecule. Water molecules and their solutes are assumed to be mobile and available to enter into chemical reactions above this point (Labuza and Altunakar, 2007). Since the calculations used in the GAB model use dry mass basis, sorption isotherms will be discussed in the dry basis—like compositional data—rather than wet basis, which is used to discuss moisture content.

## Data Analysis

Data from the laboratory storage reactors was analyzed and modeled using Excel 2011 (Microsoft Corp., Redmond, WA, United States) and JMP software (SAS Institute Inc., Cary, NC, United States). For compositional analysis (*n* = 4) and sugar release experiments (*n* = 6), single-factor one-way analysis of variance (ANOVA) was performed in SigmaPlot (version 13.0) to identify significant differences, and Tukey’s honest significant difference (HSD) test was performed if the ANOVA was significant at *p* < 0.05 for a multiple-level comparison of statistical equivalency. Sorption isotherm model parameters (*C*, *k*, and *M*_0_) were compared using the Student’s *t*-test in SigmaPlot (*p* < 0.05) to identify significant differences between the initial and stored materials’ adsorption and desorption characteristics of the GAB isotherms. When tests for normal distribution or equal variance failed a Mann-Whitney *U* test for difference between medians was used.

## Results and Discussion

### Storage Performance

**FIGURE 1:**
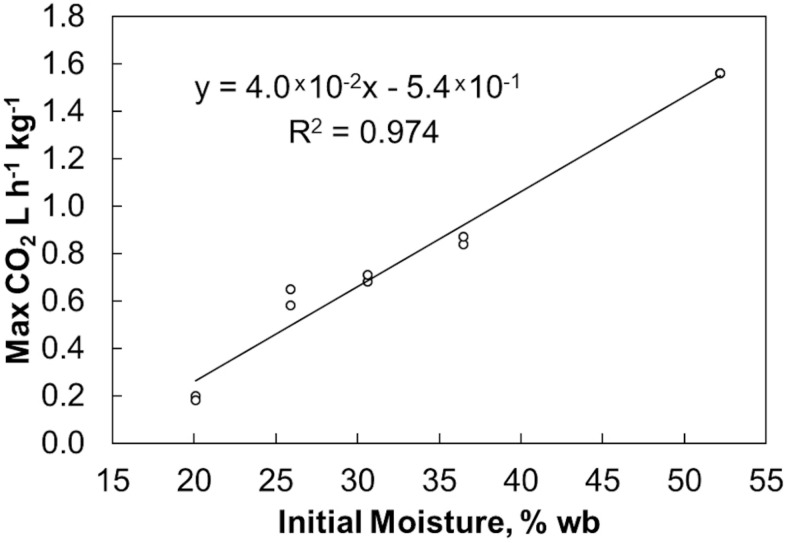
CO_2_ peaks measured in the off gas over the moisture contents used in this study.

**FIGURE 2:**
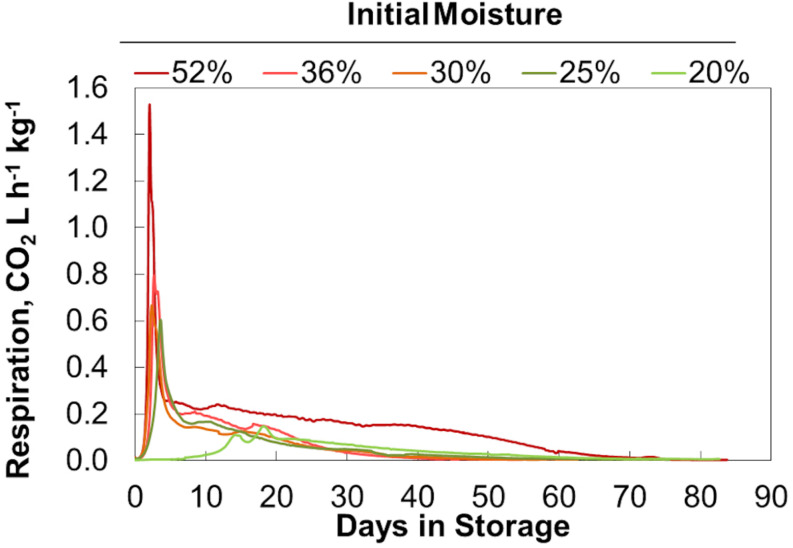
CO_2_ concentration measured in the off gas. Averages of 2 reactors at each moisture content shown.

Final bulk moisture contents remained ±5% (absolute) of the initial moisture contents. Moisture tended to migrate upwards resulting in a moisture decrease in the lower quarter of the reactors and a moisture increase in the upper quarter based on grab samples from the top and bottom (data not shown). The respiration profiles measured during biomass storage, determined by quantifying the CO_2_ released from each reactor, were proportional to the biomass moisture contents (Figure 1). Figure 2 shows that within 6 days of storage sharp CO_2_ concentration spikes can be seen in the off gas of the biomass stored between 25% and 52% moisture content. The magnitude of the respiration spike is larger and occurs earlier with increased moisture content, though the duration of the spike is similar among moistures >25%. However, the timing of the respiration pattern of the 20% moisture material differs from that of the higher moisture contents. Maximum respiration rates begin nearly three weeks later and are nearly an order of magnitude less than that of the 52% moisture conditions. Notwithstanding, the pattern of early peak respiration rate followed by lower sustained respiration rate before eventually tapering off is common among all the tested conditions. This three phase “peak, shoulder, and decline” respiration pattern is described in greater detail in Bonner et al. (2015) for both woody and herbaceous biomass storage. In summary, storage stability is greatly affected by moisture content within the first two weeks of storage. Losses are minimized at moisture contents of 20% or less. Above this level biological activity is almost immediate and is proportional to moisture content. The reasons for this delay are not clear but are likely related to the impact of water activity, as discussed below.

**FIGURE 3:**
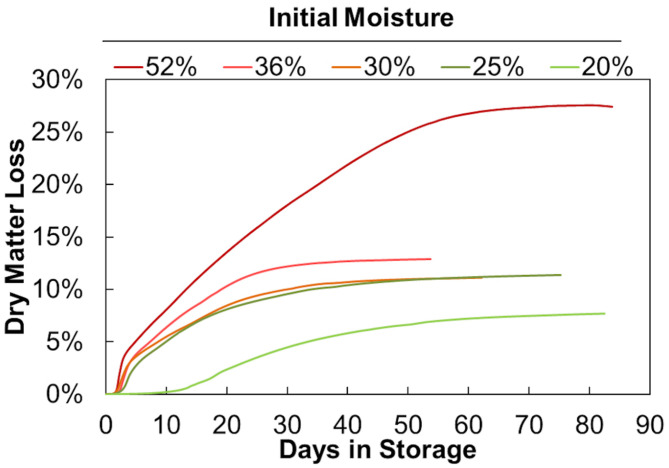
Total dry matter loss over time. Averages of 2 reactors at each moisture content shown.

Figure 3 shows the average accumulated dry matter loss over time for duplicate storage conditions. The initial loss rates (slopes) and final extents (maximum values) increase with increasing moisture content but are not linearly proportional. Onset of dry matter loss and maximum loss rates are similar for moisture contents between 25% and 52%. Onset is delayed and maximum DML rates are much less at 20% moisture. This indicates that a threshold exists between 20% and 25% moisture, above which appreciably more microbial activity occurs early in storage. Igathinathane et al. (2008) has previously shown that fungal growth on biomass is largely dependent on water activity (*a*_*w*_), with a precipitous increase in growth beginning at *a*_*w*_ values greater than 0.89. Based on their sorption experiments, corn stover at 20% moisture would have a water activity near 0.85, while stover at 25% moisture would reach >0.9, potentially traversing this critical range for microbial activity. Corn stover stored at 25%, 30%, and 36% moisture behaved similarly, both in terms of dry matter loss rate and total extent of dry matter loss. Again, this aligns well with previous work that has shown the water activity to equilibrium moisture content relationship approaches a vertical asymptote, meaning this 10% span in moisture has little effect on the water activity of the material (e.g., *a*_*w*_ 0.9 to 0.95) and presence of mold growth (Igathinathane et al., 2008). There appears to be another threshold between materials stored at 36% and 52% moisture. The 52% moisture material exhibited both a higher initial CO_2_ spike and a greater sustained rate of dry matter loss over the storage period, culminating in substantially greater total dry matter loss. However, the lack of data between 36% and 52% moisture prevents any inferences from being made as to the specific moisture content where degradation increase rapidly as a result of increasing moisture. Nevertheless, these results demonstrate that moisture reductions in the range of 36% to 25% can have a strong positive impact on aerobic storage stability.

**FIGURE 4:**
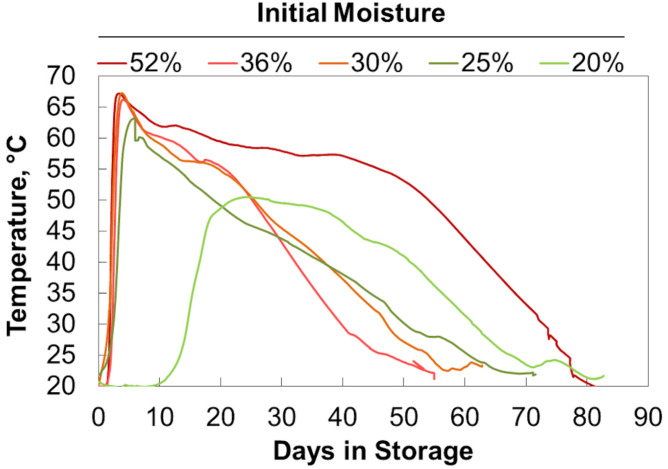
Temperature during storage. Averages of 2 reactors at each moisture content shown.

**FIGURE 5:**
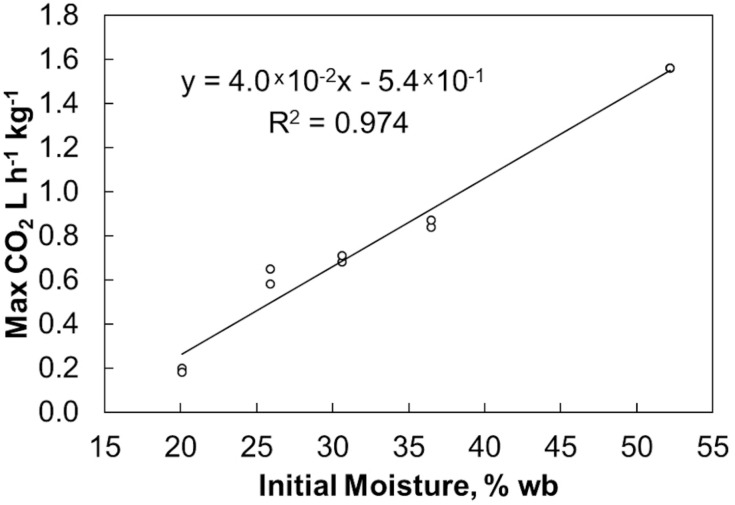
Relationship of dry matter loss to time spent at elevated temperatures. HDD represents the number of days stored material spent above 45°C.

Initial moisture content similarly affected self-heating due to microbial respiration (Figure 4). As a result of the delay in respiration seen in the 20% moisture material, these reactors exhibited delayed self-heating. After this delay, the 20% moisture samples heated to a lower maximum temperature than the other material. The 25%, 30%, and 36% moisture samples heated to similar maximum temperatures (with the 25% moisture samples having a slightly lower maximum temperature) before cooling at similar rates. Again, this matches the microbial respiration rates shown in Figure 2. The 52% moisture reactors had a heating rate and maximum temperature like those of the material ranging from 25% to 36% moisture. However, sustained microbial respiration at 52% moisture resulted in higher temperatures for a longer duration of time. Since respiration rates drive both the measured temperature and the dry matter loss, we explored the relationship between temperature, time, and extents of dry matter loss. High Degree Days (HDD) is the product of the time spent (days or fraction thereof) above a selected temperature (in this case, 45°C) and the difference between the elevated temperature and 45°C. It is a method employed by Shinners et al. (2011) for field storage and produces results in degree days above a specific temperature and indicates the severity of respiratory biodegradation in stored biomass. Plotting dry matter loss by HDD (Figure 5) shows a linear increase in accumulated dry matter loss with time spent at temperatures greater than 45°C (*r^2^* = 0.95). This relationship suggests that internal stack temperature, which can be monitored relatively easily, may be useful as a “real-time” indicator of storage stability. Specifically, internal stack temperature, which may easily be measured accurately in a number of locations, can be used to alert feedstock producers, aggregators, and end users of material instabilities that could lead to compositional changes and material losses as a result of biodegradation. This information could be used to identify lots of materials that require additional protection from moisture exposure during storage or to schedule delivery of “at-risk” materials before their conversion value is lost.

### Composition Changes

For each moisture content analyzed in the storage reactors, compositional analyses were assessed for the corn stover before and after storage. Average values are shown in Table 2. Total extractives declined in the 25%, 30%, and 35% moisture storage conditions, but they subsequently increased in the high dry matter loss conditions of the 52% reactors. Soluble glucose concentration decreased in the stored reactors relative to the initial conditions. It remained low in every tested storage condition suggesting a rapid and irreversible loss of soluble glucose, likely a result of the microbial activity occurring over time. Polymeric glucan concentration was significantly enriched in storage as noted by the *P*-value of 0.037, yet the pairwise comparisons did not detect significant differences in the means of the unstored and stored samples. The apparent increase in as-recovered glucan shown in Table 2 is a direct result of the selective loss of the hemicellulose components xylan and acetic acid (acetyl), which decrease significantly (ANOVA, *p* < 0.05 shown by superscript letters) with increasing dry matter loss. Soluble xylose increased in conditions greater than 20% moisture content, while structural xylan was significantly reduced, most notably during storage at 52% moisture. Galactan was enriched during 52% moisture storage while arabinan was relatively constant across the range of measured degradation. Acetic acid, a measure of acetyl groups within the hemicellulose, decreased because of biodegradation during storage, with over 50% reduction in the 52% moisture condition. Lignin was significantly enriched because of storage at 36% and 52% moisture, while ash was not statistically impacted as a result of storage. The changes in chemical composition resulting from dry matter loss provide insight into the impact of storage at a range of moisture contents. The enrichment of glucan at the expense of xylan resulting from dry matter loss suggests that high-moisture storage conditions lead to systematic compositional changes that may be important in conversion, especially if the process economics rely on the presence of a critical concentration of C-5 carbohydrates.

**TABLE 2:** Composition (% of dry matter) of corn stover before and after reactor storage as affected by initial moisture.

Storage	Total	Soluble	Soluble					Acetic	Total	
conditions	extractives	glucose	xylose	Glucan	Xylan	Galactan	Arabinan	acid	lignin	Ash
Unstored	10.9(1.3)^a^	2.2(0.1)^a^	0.3(0.0)^a^	34.4 (0.6)	25.3(0.7)^a^	1.5(0.2)^a^	4.0 (0.2)	4.2(0.0)^a^	11.2(0.3)^a^	4.0 (0.4)
20%	8.7(0.3)^a,b^	0.6(0.0)^b^	0.2(0.0)^a,b^	36.5 (0.9)	26.3(0.1)^a,b^	2.1(0.0)^a^	4.0 (0.3)	3.3(0.3)^b^	12.3(0.6)^a,b^	4.2 (0.5)
25%	7.7(0.3)^b^	0.7(0.0)^b^	0.3(0.0)^a,b,c^	36.7 (0.8)	25.2(0.3)^a,b,c^	2.2(0.0)^a^	4.2 (0.1)	3.1(0.3)^b,c^	11.9(0.2)^a,b,c^	4.0 (0.4)
30%	7.9(0.5)^b^	0.7(0.1)^b^	0.4(0.1)^a,b,c^	36.9 (0.7)	25.9(0.7)^a,b,c,d^	1.8(0.0)^a,b^	4.3 (0.1)	2.8(0.0)^b,c,d^	11.7(0.1)^a,b,c,d^	4.9 (0.3)
36%	7.3(1.0)^b^	0.5(0.2)^b^	0.5(0.0)^a,c,d^	36.7 (1.4)	23.8(0.2)^a,c,d,e^	1.8(0.0)^a,b^	4.0 (0.2)	2.5(0.3)^c,d,e^	12.7(0.7)^b,c,d,e^	5.0 (0.1)
52%	9.5(1.0)^a,b^	0.5(0.2)^b^	0.6(0.0)^d^	36.5 (0.3)	22.9(1.3)^c,e^	3.7(0.2)^c^	3.8 (0.1)	1.9(0.0)^e^	14.0(0.2)^b,e^	4.8 (0.0)
P-value*	0.010	<0.001	0.002	0.037	0.005	<0.001	0.178	<0.001	0.001	0.036

Values in the parenthesis represent the standard deviation; letters represent significantly different groups based on Tukey’s tests following results of an ANOVA (*p* < 0.05). *Results of ANOVA; results significant if *P* < 0.05.

### Pretreatment and Enzymatic Hydrolysis

**FIGURE 6:**
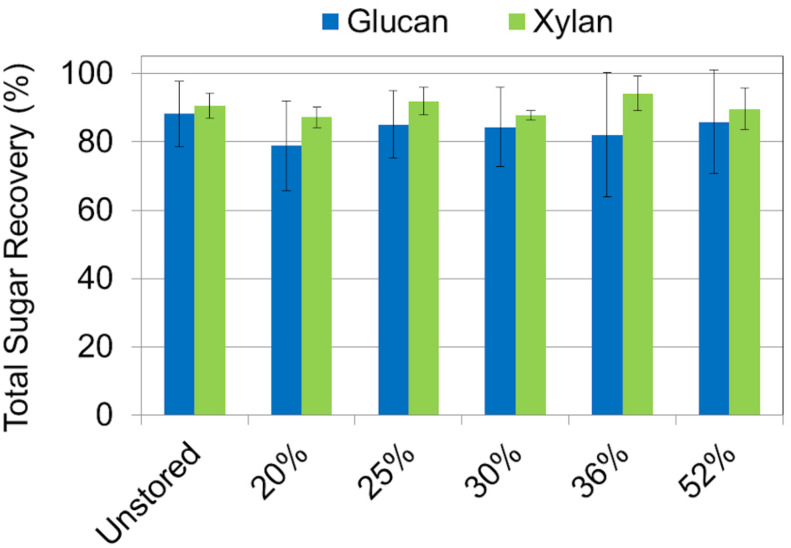
Total sugar recovery of glucan and xylan based on carbohydrate concentrations after dilute acid pretreatment, but before enzymatic hydrolysis. Results show that total glucan and xylan recovery after pretreatment were the same among all storage moistures.

**FIGURE 7:**
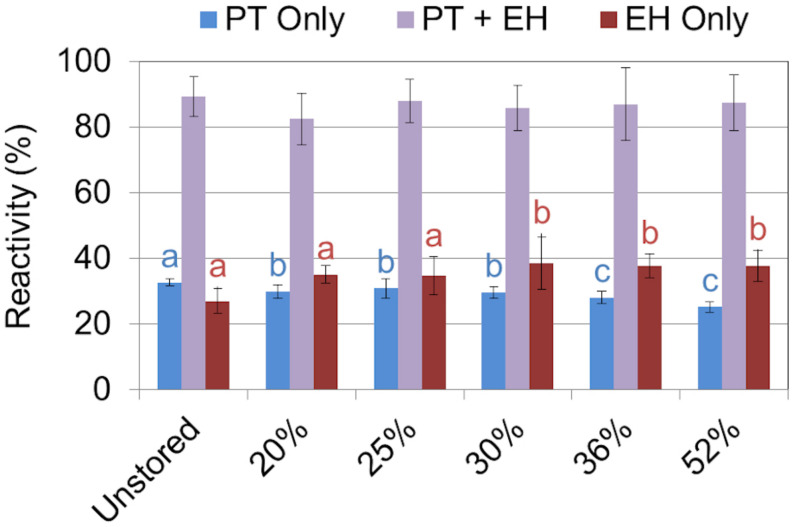
Biomass reactivity—defined as monomeric sugar released relative to total sugar initially present—as a result of dilute acid pretreatment (PT), enzymatic hydrolysis (EH), and the combination of PT and EH. Letters indicate significant differences (ANOVA, *p* < 0.05) in reactivity between the storage moistures after pretreatment (blue) and enzymatic hydrolysis (red). The combined effect of pretreatment and enzymatic hydrolysis overcame any differences seen in the individual steps alone.

Sugar recovery after combined dilute-acid pretreatment and enzymatic hydrolysis ranged from 79% to 86% of available glucose and 88% to 94% xylose being released across the range of moisture contents examined. No statistical difference in glucose or xylose yield was observed between any of the corn stover samples regardless of dry matter loss (Figure 6). The relative high severity of the dilute acid pretreatment assay used may have masked any subtle yield differences in wet harvested corn stover. Similar results have been reported in both aerobically and anaerobically stored corn stover (Wendt et al., 2018). Likewise, no difference was seen in feedstock reactivity—defined as the monomeric sugar yield relative to the total structural and soluble sugars before pretreatment—in any of the corn stover samples after combined pretreatment and enzymatic hydrolysis (Figure 7). However, statistically significant differences were seen between the sugar yields of the individual steps of dilute acid pretreatment and enzymatic hydrolysis alone. Pretreatment yields were higher for the untreated and 20% moisture content stover, intermediate for the 25% and 30% moistures, and lower for the 36% and 52% moistures, likely a result of respiratory loss of soluble sugar monomers and oligomers. A concomitant increase in structural sugar yield in enzymatic hydrolysis in the 36% and 52% moisture samples was sufficient to balance the reduction of pretreatment yields, resulting in no net change in sugar release in the combined pretreatment and enzymatic hydrolysis. These results suggest that the combined effect of self-heating and degradation increased accessibility of enzymatic attack on the remaining cellulose-rich fraction of the biomass. Degradation of hemicellulose observed in this study and elsewhere (Wendt et al., 2014, 2018) confirm these results. In summary, the differences that were exhibited in mild dilute acid pretreatment and in enzymatic hydrolysis are indicative of the minor but significant changes in structural composition as a result of high-moisture aerobic storage and associated degradation.

### Sorption Isotherms

**FIGURE 8:**
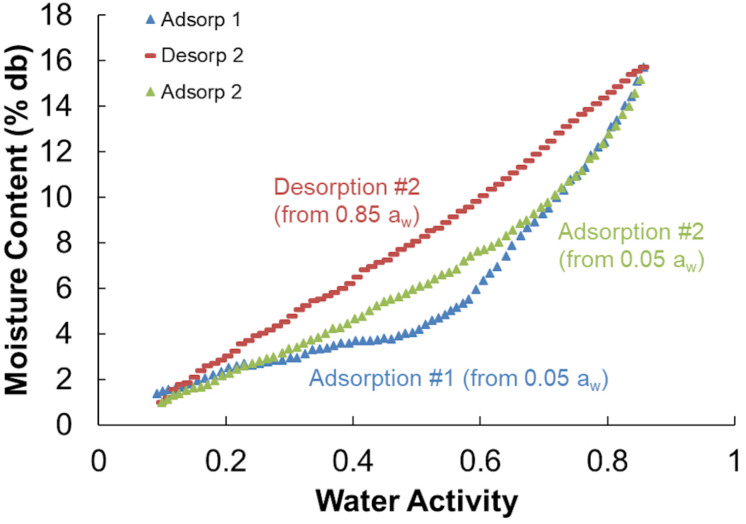
Adsorption and desorption isotherms for corn stover stored at 20% moisture content that shows the hysteresis loop used in the GAB isotherm models (Adsorption and Desorption #2) and the initial divergent adsorption (Adsorption #1).

**FIGURE 9:**
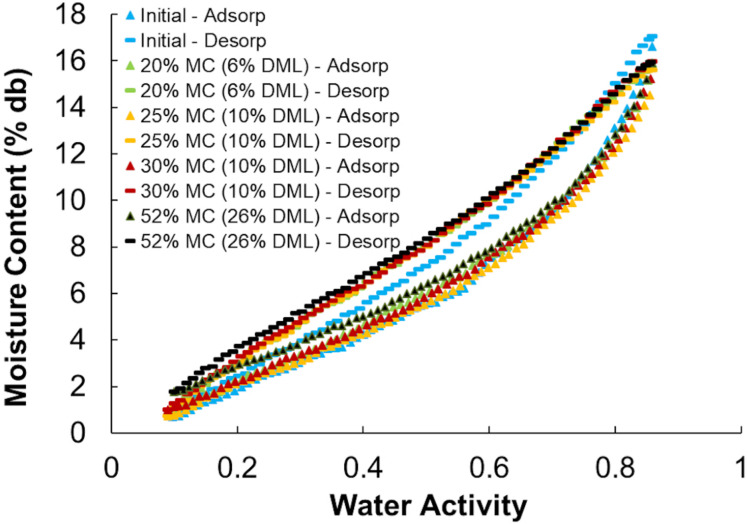
Adsorption and desorption isotherms for representative corn stover samples before (Initial) and after storage at various moisture contents.

Moisture sorption isotherms follow the sigmoidal shape of type II isotherms typical of porous biological media and other agricultural products (Igathinathane et al., 2005; Labuza and Altunakar, 2007). All isotherms showed a distinct hysteresis loop between the adsorption and desorption isotherms. The initial adsorption isotherms (Adsorption #1) were divergent from subsequent second (and greater) adsorptions (Figure 8) and because of this divergence only the second sorption cycles were used in the analyses. One representative example of each storage moisture isotherm is shown in Figure 9. Each point represents one discrete measurement of water activity at a specific weight during the test. The topmost grouping of points shows the second desorption phase. The bottommost grouping of points shows the second adsorption phase. Between them is the second desorption of the unstored material, which plots outside of the group of stored samples below it. This indicates that the unstored materials have a lower water activity (more tightly bound water; less water available for biochemical reactions) than do the stored materials at any given moisture content. For reference, 1% to 18% dry basis is equivalent to 1% to 15% wet basis and spans a range of 0.05 to 0.85 *a*_*w*_ for these corn stover samples.

Results from the individual adsorption and desorption isotherms were fit to the GAB model and the model parameters of *C, k*, and *M*_0_ were compared between unstored (initial) and the combined stored materials. This model was used as it has a viable theoretical background, is used widely in the food, forest product, and agricultural product industries, and its parameters have physical meaning in terms of the sorption process (Labuza and Altunakar, 2007). Duplicate moisture content samples were insufficient replication to evaluate more than “before” and “after” storage effects. Four initial samples were taken randomly from the original materials that went into the reactors resulting in unequal numbers of initial (*n* = 4) and after-storage (*n* = 5 and *n* = 8 for desorption and adsorption, respectively) samples tested. Insufficient sample existed to test materials from the 36% moisture reactors and three after-storage desorption isotherms were rejected as a result of analytical errors. Table 3 shows the average GAB desorption and adsorption isotherm model parameters for initial and after-storage corn stover samples. Heat constants (*C*), material drying parameters (*k*), and monolayer moisture contents (*M*_0_) were significantly altered because of storage, dry matter loss, and compositional changes noted above. Material specific drying parameters were reduced, and monolayer moisture contents were increased because of the changes that occurred during storage. The increase monolayer moisture content indicates that at the point that all sorption sites are “wetted” there is more water present in the after-storage samples than the initial samples. Possible causes for this include (1) a greater number of available sorption sites available after biodegradation, (2) the presence of more or “stronger” (more hydrophilic) sorption sites after compositional changes, and/or (3) the presence of fewer or “weaker” (less hydrophilic) hydrophobic sites blocking adsorption after compositional changes (van den Berg and Bruin, 1981). Physical and chemical changes resulting from biodegradation have the potential to open more pore spaces (loss of structural integrity), create more surface area (pitting and increased surface roughness), consume or expose biomass chemical components with different hydrophilic tendencies (van den Berg and Bruin, 1981).

**TABLE 3:** Average GAB isotherm model parameters (1-SD) for 40°C dried (∼5% mc, wb) corn stover before and after storage.

	Desorption				Adsorption			
		
	*C*	*k*	*M*_0_	*n*	*C*	*k*	*M*_0_	*n*
Unstored	2.5 (0.20)	0.77 (0.01)	7.0 (0.32)	4	3.3 (0.37)	0.9 (0.01)	3.9 (0.03)	4
After-storage	3.5 (0.46)	0.67 (0.05)	8.5 (1.05)	5	3.9 (1.2)	0.8 (0.04)	5.1 (0.74)	8
*P-value*	0.006	0.005	0.03		0.004*	0.004*	0.01	

Significant differences resulting from storage were evaluated using Students *t*-test (2-tailed) or Mann-Whitney U (for samples with unequal variances). Parameters for stored and unstored stover are significantly different for both desorption and adsorption models. Monolayer moisture contents (dry basis) are significantly higher after storage and subsequent dry matter loss. *Failed equal variance test—used Mann-Whitney U; results significant if *P* < 0.05.

## Conclusion

Variable moisture content of herbaceous crop residues at harvest impacts material stability in storage and ultimately feedstock logistics and processing performance. Despite this recognized variability, many logistics case studies rely on low moisture baled feedstock to reduce handling costs and preserve dry matter. While moisture contents above 20% risk unacceptable losses in aerobic feed and forage storage, no quantitative relationship exists between corn stover moisture content and rates or extents of degradation for bioenergy use. Without such a relationship the cost of high moisture aerobic storage, both to the producer and refinery, cannot be reliably estimated. In this work corn stover was stored using laboratory storage reactors at a range of initial moisture contents (20%, 25%, 30%, 36%, and 52%) to evaluate differences in self-heating, dry matter loss, chemical composition, sugar yield, and moisture sorption characteristics. The use of intermediate sized laboratory-scale storage reactors improved environmental control, provided high-fidelity dry matter loss measurement, and improved sampling efficiency, which reduced or eliminated some of the uncertainties associated with field and bale scale tests. Effective control, sampling, and measurements allows us to quantify the rates and extents of dry matter loss and link those losses to chemical and physical changes. The results of this study describe how storage behavior is dramatically impacted by moisture content and the resultant microbial activity, with dry matter losses ranging from 8% to 28% across the measured moisture content range. The chemical composition of these materials differed proportionately to the extent of dry matter loss, though even the most severe cases yielded quantities of total sugars comparable to fresh material when processed through dilute acid pretreatment and enzymatic hydrolysis. While the total structural carbohydrate content in the as-delivered dry matter remained relatively constant, the ratio of glucan to xylan increased significantly because of dry matter loss. When calculated on an as-harvested basis assuming a stover yield of 4.5 Mg ha^–1^ (2 tn ac^–1^) and a starting total glucan plus xylan content of 62% (2,800 kg ha^–1^), 28 kg of available sugars are lost per 1% dry matter loss occurring in storage.

Moisture sorption isotherms show that physical and/or compositional changes that occur during high moisture storage change the wetting and drying characteristics of the materials significantly. Results of this study show that short-term stability exists for corn stover stored at <35% moisture, but that above this threshold degradation is rapid and extensive. Losses occur primarily within the hemicellulose components, which result in higher as-received glucan and lignin concentrations. Dry matter loss and compositional changes measured under these controlled conditions provides the basis for predicting storage stability within a supply system that provides corn stover to a biorefinery or processing depot. Understanding biomass storage stability as a function of storage environment is necessary to develop management strategies to deliver consistent corn stover feedstock to end users. Future work will explore the details and mechanisms of the physical and chemical changes resulting from dry matter loss and examine the role that microbial communities play in these storage-related losses.

## Data Availability Statement

The datasets generated for this study are available on request to the corresponding author.

## Author Contributions

LW, IB, WS, and JM performed the storage experiments and data analysis. All authors drafted and revised the manuscript and approved the final version of the manuscript.

## Conflict of Interest

The authors declare that the research was conducted in the absence of any commercial or financial relationships that could be construed as a potential conflict of interest.
